# The Chicago Clinic

**Published:** 1902-02

**Authors:** 


### THE CHICAGO CLINIC.

The *Chicago Clinic* begins 1902, its fifteenth year, by entering a new and important field. Hereafter, as the *Chicago Clinic and Pure Water Journal,* still under the able editorial management of Drs. Marcus P. Hatfield and Geo. Thomas Palmer, it will deal mainly with such matters as pure water supply; water-borne diseases, their detection and prevention; the various mineral springs of the country, their analysis and classification; the disposal of sewage, and kindred subjects.

For many months the *Clinic* has been one of the most heartily welcomed of our exchanges. Especially have we enjoyed its editorial department. In their new and important work the editors have our best wishes.

